# Outreach, Recruitment and Engagement Cores of NIA‐designated Alzheimer's Disease Research Centers: Current strategies and future directions

**DOI:** 10.1002/alz.71238

**Published:** 2026-04-24

**Authors:** Carol K Chan, Crystal M. Glover, Monica Parker, Joshua D. Grill, Jessica B. Langbaum, Darby J. Morhardt, Ozioma Okonkwo, Dorothy Farrar‐Edwards, Sophia Wang

**Affiliations:** ^1^ Center for Brain Health Cleveland Clinic Cleveland Ohio USA; ^2^ Department of Neurology University of California, Irvine Orange California USA; ^3^ Department of Neurology Emory Brain Health Center Emory University School of Medicine Atlanta Georgia USA; ^4^ Emory Goizueta Alzheimer's Disease Research Center Emory University Atlanta Georgia USA; ^5^ Department of Neurobiology and Behavior University of California Irvine California USA; ^6^ Department of Psychiatry and Human Behavior University of California, Irvine Orange California USA; ^7^ Institute for Memory Impairments and Neurological Disorders University of California, Irvine Irvine California USA; ^8^ Banner Alzheimer's Institute Phoenix Arizona USA; ^9^ Arizona Alzheimer's Disease Research Center Phoenix Arizona USA; ^10^ Cognitive Neurology and Alzheimer's Disease Center Northwestern University Feinberg School of Medicine Chicago Illinois USA; ^11^ Wisconsin Alzheimer's Disease Research Center School of Medicine and Public Health University of Wisconsin–Madison Madison Wisconsin USA; ^12^ Department of Medicine Division of Geriatrics and Gerontology University of Wisconsin School of Medicine Madison Wisconsin USA; ^13^ Department of Psychiatry Indiana University School of Medicine Indianapolis Indiana USA; ^14^ Indiana Alzheimer's Disease Research Center Indiana University School of Medicine Indianapolis Indiana USA

**Keywords:** blood‐based biomarkers, disparities, ethnicity, higher risk populations, methods, minorities, public health, race, recruitment, registries, retention, rural

## Abstract

Populations at higher risk for Alzheimer's disease and related dementias (ADRD) are often not proportionally represented in research. These populations include but are not limited to individuals from wide‐ranging ethnic and racial backgrounds, those with lower education, and those with limited health‐care access. Addressing disparities in research participation will be essential to improving our understanding of the varied causal mechanisms, risks and protective factors, and responses to interventions to find generalizable solutions to improve health outcomes in ADRD. The goals of this perspective are to (1) review obstacles and facilitators to recruitment and retention of populations at higher risk for ADRD, (2) provide an overview of data‐driven actionable items, ongoing strategies, and proposed initiatives for the recruitment and retention of these populations in National Institute on Aging–funded Alzheimer's Disease Research Centers; and (3) highlight the roles of Outreach, Recruitment and Engagement Cores and the National Alzheimer's Coordinating Center in achieving these goals.

## Highlights

- Despite experiencing an elevated risk of Alzheimer's disease and related dementias (ADRD), higher risk populations are historically underrepresented in research.
- The Outreach, Engagement and Recruitment Core of Alzheimer's Disease Research Centers will have a central role in facilitating and evaluating the effectiveness of initiatives to improve the recruitment and retention of higher risk populations.
- Future studies should work to reduce barriers to include participants of different cultural and demographic backgrounds while taking geographical, socioeconomic, health‐care access, and social factors into account.

## 1 INTRODUCTION

Representation of populations at higher risk for Alzheimer's disease and related dementias (ADRD) within research cohorts is crucial to address health disparities and facilitate scientific advancements. Populations at higher risk include but are not limited to individuals from ethnic and racial backgrounds historically underrepresented in research, older adults who have less access to dementia specialist care based at tertiary academic medical centers due to population‐level disparities in health insurance and geographical access, and those who have comorbid medical and psychiatric disorders which preclude research participation.^1^ The National Institute on Aging (NIA) has identified fundamental indicators in their Health Disparities Research Framework, including but not limited to environmental, social, psychological, behavioral, and biological factors.^2^ Together, these factors can simultaneously contribute to a population's increased risk for ADRD and lower research participation, limiting the generalizability of research advances to populations of higher risk. Addressing the disproportionate under‐enrollment of populations at higher risk is essential to improving our understanding of causal mechanisms, modifiable risks and protective factors, responses to treatments, and finding solutions to reduce the public health burden of ADRD. Identifying and understanding these nuances will play a key role as the field moves toward precision medicine, an approach for disease prevention and treatment personalized to an individual's specific pattern of biological variability, environmental factors, lifestyle factors, and psychosocial factors.^3^ To do so, the field must improve our understanding of the personal nuances in decision making and daily practices that impact health disparities and participation in research and develop tailored methods to address health disparity considerations in research.^4^

## 2 THE ROLE OF OUTREACH, Recruitment and engagement coRES IN THE RECRUITMENT AND RETENTION OF POPULATIONS AT HIGHER RISK

**FIGURE 1:**
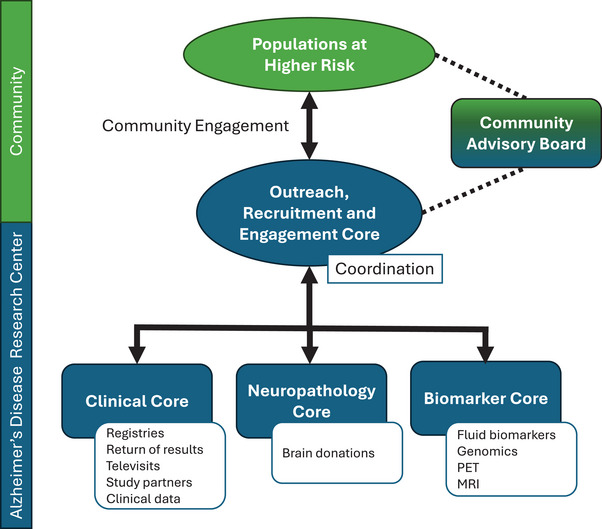
Interaction among the Outreach, Recruitment and Engagement Core; other cores of Alzheimer's Disease Research Centers; and populations at higher risk. The Administrative Core and Data Management and Statistical Core are not shown. White boxes represent the role of each depicted core as it relates to interaction with participants. MRI, magnetic resonance imaging; PET, positron emission tomography.

There are currently 35 Alzheimer's Disease Research Centers (ADRCs) at major medical and academic institutions across the United States. They consist of multiple “cores” that work collaboratively to conduct research on ADRD. Key components include the Administrative Core; Clinical Core; Data Management and Statistical Core; Neuropathology Core; Biomarker Core; and Outreach, Recruitment and Engagement Core (OREC). ORECs play an essential role as a liaison among ADRCs, older adults, people living with dementia, their care partners, and the community (Figure 1). Their role is critical to improving the involvement of understudied populations and populations at higher risk of ADRD in research and reducing disparities in research representation by developing, implementing, and evaluating strategies to reach recruitment goals.

Participants enrolled in NIA‐designated ADRCs provide data for one of the largest ADRD datasets in the world: the Uniform Data Set (UDS) available through the National Alzheimer's Coordinating Center (NACC; naccdata.org). The NACC Data Platform includes standardized data on > 50,000 participants, with > 17,000 actively followed. Recruitment of higher risk populations for the NACC has unique challenges that may introduce a selection bias, including the use of convenience sampling, relatively intensive participant requirements, and the requirement of access to tertiary medical centers. A selection bias occurs when the sample selection does not accurately reflect the target population and can jeopardize the validity of estimating the relationship between variables. Homogenous and select sampling can pose barriers to understanding the varied causal mechanisms, risks and protective factors, and responses to interventions to find generalizable solutions to improve health outcomes in ADRD. While substantial efforts have been made to increase the representativeness of the ADRC cohorts, a recent study found that participants enrolled at ADRCs within this network may not be representative of the country's population in key demographic measures.^5^ NACC participants were older, had more years of education, worse subjective cognition, and less self‐reported depressive symptoms than participants from the Health and Retirement Study, a longitudinal cohort of community‐dwelling US adults aged ≥ 50 and their spouses.

**FIGURE 2:**
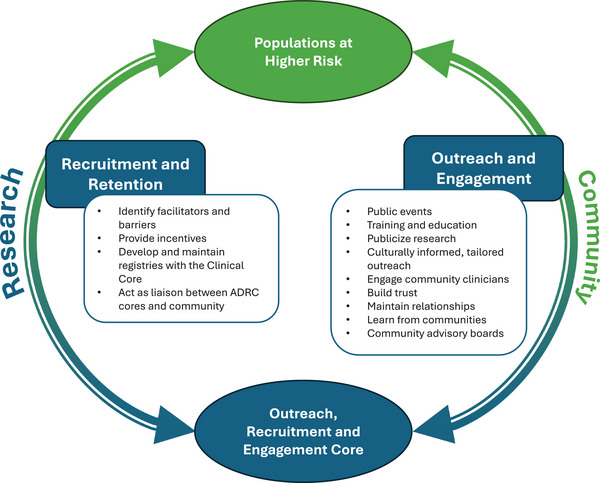
Activities of the Outreach, Recruitment and Engagement Core in the recruitment, retention, outreach, and engagement of populations at higher risk. ADRC, Alzheimer's Disease Research Center.

Health disparities stem from systemic policies and practices which lead to disparate outcomes: preventable differences in disease burden and opportunities to achieve optimal health. Elimination of health disparities is attained when everyone has a fair and balanced opportunity to reach their highest level of health.^6^ ORECs have the unique opportunity to reduce research and health disparities in ADRD through improving the proportional representation of study cohorts at ADRCs relative to the general population. Key OREC activities include public‐facing events, providing training and education, developing tailored outreach, and direct engagement of community groups to encourage recruitment and retention of participants. These activities provide a framework on which ORECs can continue to build upon, to develop strategies for reducing research disparities in ADRD (Table 1) and to continue fostering bidirectional relationships between researchers and the community (Figure 2).

**TABLE 1:** Strategies and actionable items for improving recruitment of populations at higher risk to Alzheimer's disease and related dementias studies.

Strategy	Recommendations
***Strengthen partnerships and alliances***	Develop bidirectional community input through community advisory boards Acknowledge past institutional wrongdoings Have culturally congruent researchers Leverage relationships with clinicians and primary care providers in the community Leverage opportunities for timely dissemination for research findings during community outreach Include social networks, including families and coordination with personal physicians
***Expanding knowledge***	Provide linguistically relevant outreach Develop accessible and culturally informed education materials on research participation Provide culturally informed training for researchers Provide options for disclosure of research results Develop education materials clearly detailing the purpose, process, and expected outcomes of procedures
***Improve research programs and policies***	Use registries for individuals who may have screened out of other studies but have self‐identified as being interested in research Adjust technological strategies to meet the population's level of digital access Allow for more flexibility in study partner criteria
***Reduce structural and environmental barriers***	Provide financial reimbursements for costs associated with study participation Allow for virtual or mobile screening Consider the use of televisits Consider providing study partners with caregiver support, reimbursements for travel and lost labor, case management Develop methods for specimen collection that can take place in the community

In this article, we summarize the recent literature on obstacles and facilitators in recruitment and retention of understudied populations, explore opportunities and actionable items for improving recruitment and engagement, and identify ways ORECs can be anticipated to contribute to these changes in the future.

## 3 OBSTACLES AND FACILITATORS TO RECRUITMENT

To date, most studies on obstacles and facilitators in the recruitment of populations at higher risk have focused on minority racial and ethnic groups.^7^ Thus, the factors described in this section may not apply to all populations at higher risk. Additional work is needed within local communities to understand obstacles and facilitators that apply within specific and personalized contexts.

### 3.1 Exclusion due to medical comorbidities

Many ADRD studies, including ADRCs, exclude people with comorbidities that may act as confounders, including cerebrovascular disease, psychiatric conditions, and medical comorbidities.^8^ These comorbidities are more common in individuals at higher risk of ADRD, including those from racial and ethnic minority groups, and those with lower income and lower education.^1^ Thus, the use of overly restrictive criteria may contribute to the exclusion of groups at highest risk of ADRD.

### 3.2 Hesitancy toward research and medical staff

There is a well‐documented history of ethical violations in research, which is often cited as a contributor to distrust from potential participants. However, older adults from understudied populations may also have additional experiences that contribute to distrust, such as the perpetuation of structural disparities by health‐care institutions and experiences of structural prejudice in their pursuit and receipt of health‐care services, thus contributing to concerns about unfair treatment in research. For example, a survey of older adults from understudied populations found that the top factors that increased the likelihood of participation included having trust that the research was fully explained to them and that researchers were honest about risks.^9^

### 3.3 Limited engagement and access

Understudied populations are willing to participate in and desire more information about ADRD research involvement,^10^, ^11^ but face obstacles in access. Many factors, including stigmatized beliefs about dementia,^10^ low willingness to disclose memory problems to health‐care providers,^12^ and lower rates of diagnosis despite evidence of impairment,^13^ contribute to fewer referrals by health‐care professionals of higher risk and understudied populations to ADRD studies than populations at lower risk.^14^ Structural factors such as lack of access to reliable transportation, research schedules that don't accommodate work and family demands, and lack of access to high‐speed internet and devices that can facilitate video visits may also limit their ability to participate in studies.^15^, ^16^

### 3.4 Participant burden

Participation in clinical studies requires not only effort and time from participants. A study at the Michigan ADRC found that participants who identified as Black or African American reported significantly higher levels of perceived research burden relative to participants who identified as non‐Hispanic White, and that participants with a dementia diagnosis reported higher levels of burden than cognitively unimpaired participants.^17^ Beyond the indirect “costs” or “burden” to participants, concerns about direct financial costs such as travel costs or costs related to lost wages are potential barriers to recruitment, particularly for understudied populations.^18^, ^19^

### 3.5 Language barriers

The American Community Survey found that, among older adult immigrants in the United States, only 44.6% spoke English “very well.”^20^ Limited opportunities to participate in preferred languages and lack of information about ADRD and study participation presented in potential participants’ preferred languages pose limitations to outreach and recruitment.^21^, ^22^ Many ADRD studies include ineligibility criteria related to language and literacy.^22^ These challenges are of particular concern considering that individuals with lower education and literacy are at greater risk of dementia; these challenges further exacerbate the exclusion of this high‐risk population.^23^

## 4 PARTICIPATION IN PROCEDURES

Procedures measuring disease‐specific biomarkers, such as lumbar punctures (LPs), positron emission tomography (PET), and brain donations are of particular interest to ADRCs. Broad participation in research procedures, particularly for biomarker characterization, is essential to achieving fair access to the latest advances in ADRD. However, our understanding of the unique obstacles and facilitators of participation in these research procedures is limited. In a survey of community‐based recruitment of registry participants, populations at higher risk were less willing to be contacted for studies requiring LPs, PET scans, and brain donation.^24^ These procedures are often perceived as more invasive and riskier than blood draws, magnetic resonance imaging scans, and cognitive testing, for which no differences in willingness to be contacted have been observed in populations at higher risk. Hence, we will focus on obstacles and facilitators to the participation of understudied populations in LPs, PET scans, and brain donations.

### 4.1 Lumbar Punctures

Some studies suggest that populations at higher risk are more likely to express concerns about LPs, citing the requirement of LP as a primary reason for non‐participation in research.^25^ Ideal LP research scenarios described by potential participants from populations at higher risk included studies that had financial incentives, and provided full disclosure of results.^26^ The desire for result disclosure is echoed in another study that found that participants who believe that the LP will result in helpful information are more likely to agree to participate.^27^

### 4.2 PET brain imaging

The literature on facilitators and obstacles in the recruitment of populations at higher risk to studies with PET brain imaging is limited. Participants from populations at higher risk have reported that altruism and positive prior experiences with research were facilitators of participation.^28^ However, participation was largely dependent on participants’ understanding of the purpose, process, and outcomes of PET brain scans. Obstacles to participation largely pertained to a lack of information about or unfamiliarity with the procedure.^28^ For example, participants oftentimes spoke of PET brain scans in conjunction with other imaging modalities or procedures such as brain donation and did not know what to expect in terms of the process of obtaining the scan. Social networks were particularly important to participants’ decision‐making process. Many planned to seek advice from their loved ones, expressed a desire to speak to another older adult who had taken part in a PET brain scan, and to speak with their personal health‐care provider for more information, especially regarding potential side effects and any associated risks.

### 4.3 Brain donation

Research participants from populations at higher risk have expressed a desire for more information about brain donations.^29^, ^30^ Positive predictors of participation in brain donation in this group include having an understanding of how the brain was used by researchers, and understanding what needed to be done to ensure their brain would be donated, while concerns that researchers may not be respectful of the body during autopsy was one of the top negative predictors.^29^ Participants have also reported a desire for more information about what was needed to carry out the brain donation process and for the results to be returned.^29^, ^30^

The lack of information is not limited to participants. Health professionals have also reported a lack of access to education materials to support discussions on brain donation.^21^ Of those who did have access to materials, most did not have access to Spanish‐language materials. Health‐care professionals were also not comfortable discussing the process of brain donation, including the removal of the brain, what was done with the brain once removed, what one needed to do ahead of time to make sure the brain is donated after a person dies, and how to talk to family members about the desire to donate one's brain.

## 5 RETURN OF RESULTS

Multiple studies examining obstacles and facilitators to recruitment have identified a desire for participants to know the results of their tests,^31^ particularly in populations at higher risk.^26^, ^28^ For studies that involved LPs, participants preferred studies that provided full disclosure of research results,^26^ and were more likely to participate in LPs if they felt there would be useful information for improving their health.^27^ Participants considering studies involving PET brain scans have also expressed that they were interested in the test results as a primary motivator for participation, and planned to share the results of their scan with their social network, including their personal doctors.^28^

A 2019 survey found that most ADRCs return results related to cognitive status and diagnosis, but not biomarker testing. Only 43% return amyloid PET results, 10% return tau imaging results, and 7% return apolipoprotein E (*APOE*) genotype results.^32^ The most frequently cited reasons for not disclosing certain biomarker and genetic findings included: the tests used did not meet clinical standards, the study consent had explicitly noted that results would not be returned, and results were not medically actionable. Since the 2019 survey, guidance for disclosures has been developed by the NACC for ADRCs.^33^ The guidance provides a framework from which ADRCs can develop their own protocols for disclosure, and stresses that the persons performing disclosures must have the expertise to answer questions about the meaning and outcomes and to assess for potential participant reactions and needs.

There is an emerging body of literature on the process and impact of disclosing genetic and biomarker risk information for ADRD. While there has been work on *APOE* genotyping and amyloid neuroimaging disclosures, the literature on other genetic and biomarker information such as polygenic risk scores, tau imaging, and blood‐based biomarkers (BBMs) is at an emerging stage.^32^

## 6 PARTICIPANT RETENTION

ADRD studies require significant time and resources from participants, particularly in longitudinal studies. Loss to follow‐up in research disproportionately affects populations at higher risk, and can affect the internal and external validity of a study, creating a selection bias.^34^

Ensuring that studies align with participant values is key to retention. Participants who feel that the study helps them fulfill their personal goals have higher retention and higher attendance to study visits.^31^ ADRCs have successfully improved retention of participants across demographic and diagnostic groups by maintaining a diverse staff, engaging staff in regular retention trainings, ensuring that specific staff members work with specific participants over time, and effective communication of study requirements and details.^35^ Staff training strategies included enhancing study description, communication of study requirements and details, communicating expectations for study participation, and helping participants understand and plan for study requirements during the recruitment and informed consent process.^35^

## 7 STUDY PARTNERS

Most ADRC studies require study partners, which may pose an obstacle to enrollment, particularly for understudied populations and populations at higher risk. A retrospective analysis of the Alzheimer's Disease Cooperative Study found that most participants who enrolled with study partners do so with a spouse (67%),^36^ with 26% enrolling with adult children as their study partners. Understudied populations are less likely to enroll with a spousal study partner.^37^ These variations may contribute to a selection bias and under‐enrollment of populations at higher risk. Participants without a spousal study partner may also face difficulties finding someone who satisfied the requirements of the study partner role,^37^ or face difficulties in coordinating study visits if their prospective study partner is still in the workforce or has family responsibilities.

## 8 ACTIONABLE STRATEGIES

### 8.1 Improving outreach and trust

In their community outreach roles, ORECs fill a critical function in building trust between ADRCs and communities. Strategies for building trust, particularly with communities at higher risk, include addressing past wrongdoings upfront, examining if problematic relationships between a particular institution and the community exist, building trust to overcome past wrongdoings, assessing if certain opportunities have not been brought to a particular community because of assumptions made about the community as a whole, and earning the trust and endorsement of organizations with longstanding ties to the community (e.g., local health centers, churches).^7^, ^11^, ^38^ Researchers can also demonstrate their concern toward the community by updating materials in languages accessible to potential participants, having researchers from congruent backgrounds, promoting research protocols tailored to their values, and having a physical and invested presence in communities from which they are recruiting.^11^ Building trust in the community takes consistent effort over time. This should be taken into consideration when allocating resources and evaluating the efficacy of intervention outcomes.

### 8.2 Community‐informed outreach

Qualitative studies with participants from understudied communities and communities at higher risk have emphasized the importance of culturally and linguistically relevant outreach, including the availability of recruitment materials in their preferred language, using culturally specific media spaces, and conducting engagement events at culturally specific spaces.^11^, ^39^ When developing outreach strategies, investigators should strive to use a communication framework, such as the Reasoned Action Approach (RAA),^40^ to develop evidence‐based targeted recruitment messages. RAA‐based communication strategies are designed to reinforce the beliefs that are positively associated with intention and counter‐argue the beliefs that are negatively associated with intention, and have been used to develop messages for many health behaviors, including recruitment of populations at higher risk into ADRD registries.^41^

### 8.3 Collaborator and community engagement

Community advisory boards (CABs) are an effective way to engage community stakeholders in research recruitment. CABs serve as liaisons between ORECs and community members (Figure 1). The National Institutes of Health ADRD Clinical Studies Recruitment Planning Guide recommends the establishment of CABs to develop and maintain trust between community stakeholders and research teams. They recommend recruiting members who reflect the heterogeneity of the local community and catchment area, identifying board members who serve as community leaders, and considering including a research participant and/or caregiver on the board to provide a personal perspective.^42^ Successful implementation of CABs has been described by several groups for facilitating outreach and increasing enrollment of understudied populations in ADRD studies, including but not limited to older adults from minority racial and ethnic groups and LGBTQIA+ older adults.^43^, ^44^

### 8.4 Expanding recruitment strategies

#### 8.4.1 Engaging primary care providers

Primary care providers are well placed to identify patients who may be at higher risk for developing AD and who may be interested in clinical trials.^45^ AD is frequently diagnosed in primary care settings, as opposed to specialty clinics. Increasing education and outreach to primary care physicians to increase knowledge about trials and referrals can help facilitate recruitment to ADRD studies, and has been successfully implemented to increase rural research participation.^46^ Primary care providers often have longstanding relationships and trust with patients and may be considered by some to be part of their social network. Studies of older adults from populations at higher risk have found that participants valued the input of their personal doctors^28^ and preferred recruitment through a physicians’ office, citing personal connection as a motivating factor.^26^

Advances in BBMs for AD are a major step in democratizing AD diagnostics, with the potential for improving access to biomarker diagnosis outside of specialized centers. The US Food and Drug Administration (FDA) recently approved the use of plasma phosphorylated tau181 for use in primary care settings to rule out AD in adults aged ≥ 55. However, widespread use without careful consideration of other clinical variables that may affect the performance of AD BBMs could have unintended negative consequences such as misinterpretation of results, misdiagnosis, and mismanagement. While referrals to specialists may mitigate some of these concerns, the shortage of dementia subspecialists is well documented.^1^ Referrals without appropriate triaging could further lengthen the already prolonged wait times. Furthermore, the need to regularly refer patients for AD BBM interpretation would significantly reduce their potential to improve access to accurate AD diagnosis in the community. ORECs have an opportunity here to expand on their role in training and education to primary care providers. This could have a dual impact on both clinical care and strengthening recruitment pathways from representative community‐based samples. The Georgia Memory Net^47^ is an example of successful implementation of a network integrating research partners with providing support for patients and their primary care providers.

#### 8.4.2 Digital strategies

Expanding the use of digital strategies may facilitate recruitment of populations at higher risk by expanding geographical reach and reducing study visit burden. The Alzheimer's Disease Neuroimaging Initiative (ADNI4) Digital Study has demonstrated that the use of a digital approach to recruitment and screening, in which participants were recruited using digital advertising and completed digital surveys and cognitive screening tests, was both feasible and effective in recruiting participants from populations at higher risk.^48^ Considerations for integrating technology to approach recruitment challenges include the use of digital screening strategies, such as combining outreach campaigns with digital self‐assessment tools that could be used to detect cognitive impairment and refer to local trial sites, memory clinics, or dedicated registries and using computerized cognitive assessments and video conferencing for study visits.^45^ Investigators should consider using digital strategies in tandem with traditional strategies to account for disparities in technological access. More work is needed to assess the effectiveness of these strategies in translating recruitment of populations at higher risk to completion of in‐person visits and biomarker assessments.

#### 8.4.3 Registries

Registries of candidate participants can aid in accelerating recruitment to ADRD studies. Most registries include data on demographics, cognitive status, and functional status, though data on caregiver burden, medication use, and health‐care use have been identified as critical gaps in existing registries.^49^ When paired with community outreach, they have been effective in the recruitment of understudied populations and in expanding the geographical reach of ADRCs.^45^ Registries could further be used to engage people who have self‐identified as being interested in research participation, but were not enrolled in trials due to screening failure.^45^ Finally, electronic medical records may also provide opportunities to target specific groups for registries.^38^, ^45^

Online enrollment has also been successfully used for registry recruitment. The Brain Health Registry, an online registry, has successfully used strategies such as multilingual support, flexible biofluid collection methods, and payments for task completion to increase their enrollment of populations at higher risk.^50^ Understudied populations are more likely to use smartphones when seeking health information, and are less likely to have a traditional computer or home broadband.^51^ Thus, the use of online recruitment materials formulated for mobile device access may help bridge the gap in enrollment to registries for populations at higher risk.^51^

Other methods of promoting registries have also been found to be effective in the enrollment of populations at higher risk, and investigations into recruitment preferences could further inform tailored recruitment strategies. For example, the Collaborative Approach for Asian American and Pacific Islander (AAPI) Research and Education (CARE) registry, the first known research registry for AAPI individuals, found that participants preferred learning about the registry from the health‐care community and hearing about it from social media or instant messaging, as opposed to flyers or workshops and seminars.^52^

Registries could also provide a valuable opportunity to provide education and provide enrollees with positive research experiences, thus increasing their likelihood of converting registrants to study participants.

### 8.5 Participation in ADRD studies with procedures

#### 8.5.1 Improve educational materials

ORECs, in collaboration with ADRC Clinical Cores, should continue to develop accessible education materials. Printed education materials could help facilitate participation by allowing participants to review the information again on their own time, serving as a point of reference when discussing the decision with their social networks.^28^ Educational materials should include the purpose of the procedure, particularly in relation to aging research; the process of the procedure; and the expected outcomes of the procedure in relation to research and to the participants. They should be written at an accessible reading level, and available in multiple languages.

#### 8.5.2 Family‐centered approach

Studies have referenced participants’ desire to discuss participation with their families.^29^, ^30^ For brain donation in particular, family input can be very important to participants. Highlighting the benefits of brain donation using a family‐centered approach could make participation more approachable, provide education on the process of brain donation and its role in research, and increase engagement.^53^

#### 8.5.3 Health‐care professional training

Given that the need for more information is a frequently cited obstacle to procedure participation,^28^, ^29^, ^30^ increased education of health‐care professionals in the process of study procedures is needed to improve trust and facilitate conversations between health‐care professionals and potential participants.

### 8.6 Reduce participant burden

Virtual study visits are one way to help reduce participant burden and facilitate visits for participants who do not live near ADRCs. While successful models of recruitment of populations at higher risk using telephone and online method have been described,^48^ more work on its effect on retention is needed. During the COVID‐19 pandemic, ADRCs had to adjust their methodologies to allow for virtual assessments, providing a roadmap of how technology may be used to reduce participant burden. The use of remote assessments also has the potential to provide more flexibility for non‐spousal study partners, non‐cohabitating study partners, or study partners who are still working. Investigators should also, however, consider the accessibility of technology in the populations they are studying to avoid selection bias due to disparities in internet access, device ownership, and comfort with technology use.

Several qualitative studies have observed that participants prefer research studies that offer financial incentives.^11^, ^26^, ^28^ One study with a large sample of populations at higher risk found that modest levels of renumeration increased willingness to participate across racial, ethnic, and income groups; had the effect of lowering perceived burden; and did not affect perceived risks or altruistic benefits.^18^

Financial incentives do not have to be limited to monetary rewards and reimbursement of travel costs. Creative monetary incentives, such as supplementing monetary reward with compensation for lost labor to participate in research, free medical care while engaged in the study, free health‐care services,^11^ reimbursement for time spent on completing pre‐screening activities, and for hiring caregivers for respite care^4^ could both provide incentives and reduce participant burden. Investigators should work with their institutional review boards to ensure ethical use of financial incentives, particularly when working with vulnerable populations (i.e., those with cognitive impairment). The NACC currently has remuneration guidelines for ADRCs, addressing the different functions of payment including reimbursing, compensating, and incentivizing participants.^54^

Sharing results with participants and the community may also provide incentive for retention.^38^ ORECs should leverage opportunities for timely dissemination of research findings. Local educational presentations, flyers, and social media could increase awareness and have the additional benefit of appealing to the altruistic motivations of participants, to show that their contributions have been meaningful.^11^

Another way to reduce participant burden is to find ways to encourage participation of study partners. Study partners play an important role in ADRD research as knowledgeable informants, providing information about a participant's cognition, function, and response to intervention. Adjustments in the criteria for study partner participation, such as allowing participants to have multiple care partners or allowing participants to attend some research visits alone may reduce impediments to research participation.^55^ A recent NACC survey of study partner burden that recruited a sample of study partners from four ADRCs found that barriers to retention included limited information about study findings, fatigue, inconvenient travel, distance, physical pain, breach of privacy, long visits, emotional distress, and difficulty keeping track of procedures.^56^ Given that many obstacles cited by study partners relate to participation burden, steps to alleviate these burdens may encourage their participation. For example, providing caregiver support and education, financial compensation, and reimbursement for costs related to travel and respite care^4^ could help reduce burden.

## 9 FUTURE DIRECTIONS

To date, most studies on the recruitment and retention of understudied populations and populations at higher risk of developing ADRD have focused on differences across minority racial and ethnic groups.^7^ While this is an important component, the study of other factors that make up the biological, psychological, and social factors of health is needed to develop an individualized framework from which tailored recruitment strategies for ADRD studies can be built. Future studies should not only strive to include participants of different cultures, identities, and demographics, but also consider other aspects of personalized nuances such as geographic, social, and economic status; health‐care access; and social factors. They should, in their recruitment and retention plans, account for social determinants of health such as access to health care, access to transportation, access to technology, personal values, social and economic status, and caregiver availability. Further study into these factors is needed to develop tailored recruitment strategies for populations at higher risk. In addition to traditional strategies used by ORECs such as linguistically appropriate materials, CABs, and community outreach, novel strategies for the development of registries, use of technology for recruitment and study visits, and creative incentives could be considered. Beyond the implementation of these strategies, studies assessing their effectiveness are needed to provide high‐quality evidence on best practices to improve the involvement of populations at higher risk in ADRD research. Adequate funding and allocating resources to support the time of participants, CABs, and outreach activities are key to furthering these goals.

The NACC UDS provides standardized longitudinal neurocognitive data collection across ADRCs. UDS 4.0 was launched in 2025, including new forms focused on social determinants on health, and more comprehensive questions on sex identity, sexual orientation, and multi‐racial and multi‐racial ethnic identities. Spanish and Chinese translations of forms are also in progress. These new initiatives are well positioned to answer critical questions about cohort heterogeneity in research, as well as testing data‐driven strategies for recruitment and retention in ADRCs. With the introduction of UDS 4.0, ADRCs will collect information related to social determinants of health, including transportation, financial security, social connections, environment, and experiences with the health‐care system. These data will allow investigators to further their understanding of how these factors affect various aspects of ADRD, for ORECs to expand their work in facilitating proportionate representation of populations at higher risk at ADRCs, and to improve generalizability of research advances.

Finally, the introduction of AD BBMs provides opportunities not only for more accessible, decentralized screening but also new ways for ORECs to engage primary care providers in research. ORECs can expand their role in community education by providing primary care providers with training on how to use and interpret AD BBMs. This type of health‐care provider engagement has potential to not only improve clinical care but also develop new recruitment pathways for individuals from higher risk groups who are less likely to have access to tertiary specialist centers.

## CONFLICT OF INTEREST STATEMENT

J.D.G. reports grants from the NIA (AG066519), Alzheimer's Association, BrightFocus Foundation, Lilly, Biogen, Genentech, and Eisai and personal fees from SiteRx. J.B.L. reports receiving grants from NIA (1UF1AG046150, RF1AG041705, R01AG05544, 1R01AG058468, and P30AG072980) and received consulting fees from Alector, Biogen, Denovo Biopharma, and Provoc. O.O. has received consulting fees from Mayo Clinic Rochester and IUPUI, and holds a leadership or fiduciary role in the International Neuropsychological Society (Board Member) and previously held an advisory role for Society for Black Neuropsychology. Other authors report no competing financial interests to declare. Author disclosures are available in the supporting information.

## FUNDING INFORMATION

This project was supported by grants from the National Institutes of Health/National Institute on Aging: P30AG072959 (C.K.C.), P30AG072976 (S.W.), 5P50AG016573 (C.M.G.), P30AG066511 (M.P.), P30AG072980 (J.B.L.), P30AG066519 (J.D.G.), P30AG013854 (D.J.M.), P30AG062715 (O.O., D.F.E.).

## Supporting information



Supporting Information
