# Supplementary material for: Outreach, Recruitment and Engagement Cores of NIA‐designated Alzheimer's Disease Research Centers: Current strategies and future directions
Source: Alzheimers Dement. 2026 Apr 24;22(4):e71238. doi: 10.1002/alz.71238 (PMC13107418; doi:10.1002/alz.71238)
Supplement: Supplementary file 1 — Supporting Information [file ALZ-22-e71238-s001.pdf]

ICMJE DISCLOSURE FORM

**Date:**	1/15/2026
**Your Name:**	Jessica Langbaum
**Manuscript Title:**	Outreach, Recruitment and Engagement Cores of NIA-Designated Alzheimer's Disease Research Centers: Current Strategies and Future Directions
**Manuscript Number (if known):**	ADJ-D-25-03572

In the interest of transparency, we ask you to disclose all relationships/activities/interests listed below that are related to the content of your manuscript. "Related" means any relation with for-profit or not-for-profit third parties whose interests may be affected by the content of the manuscript. Disclosure represents a commitment to transparency and does not necessarily indicate a bias. If you are in doubt about whether to list a relationship/activity/interest, it is preferable that you do so.

The author's relationships/activities/interests should be defined broadly. For example, if your manuscript pertains to the epidemiology of hypertension, you should declare all relationships with manufacturers of antihypertensive medication, even if that medication is not mentioned in the manuscript.

In item #1 below, report all support for the work reported in this manuscript without time limit. For all other items, the time frame for disclosure is the past 36 months.

		**Name all entities with whom you have this relationship or indicate none (add rows as needed)**	**Specifications/Comments (e.g., if payments were made to you or to your institution)**
		Time frame: Since the initial planning of the work	
1	All support for the present manuscript (e.g., funding, provision of study materials, medical writing, article processing charges, etc.) No time limit for this item.	☐ None	
		NIH (P30AG072980)	Institution
			Click the tab key to add additional rows.
		Time frame: past 36 months	
**2**	Grants or contracts from any entity (if not indicated in item #1 above).	☐ **None**	
		NIH	Institution (NIH grant numbers R01AG086363, R33AG070604, R01AG069453, R01AG063954, R01AG058468)
		Eli Lilly	Institution
		Roche	Institution
**3**	Royalties or licenses	☒ **None**	

		Name all entities with whom you have this relationship or indicate none (add rows as needed)	Specifications/Comments (e.g., if payments were made to you or to your institution)
**4**	Consulting fees	☐ **None**	
		Denovo Biopharma	self
		Premiere Inc	self
**5**	Payment or honoraria for lectures, presentations, speakers bureaus, manuscript writing or educational events	☒ **None**	
**6**	Payment for expert testimony	☒ **None**	
**7**	Support for attending meetings and/or travel	☒ **None**	
**8**	Patents planned, issued or pending	☒ **None**	
**9**	Participation on a Data Safety Monitoring Board or Advisory Board	☒ **None**	
**10**	Leadership or fiduciary role in other board, society, committee or advocacy group, paid or unpaid	☒ **None**	

		Name all entities with whom you have this relationship or indicate none (add rows as needed)	Specifications/Comments (e.g., if payments were made to you or to your institution)
**11**	Stock or stock options	☒ **None**	
**12**	Receipt of equipment, materials, drugs, medical writing, gifts or other services	☒ **None**	
**13**	Other financial or non-financial interests	☒ **None**	

Please place an "X" next to the following statement to indicate your agreement:

☒ I certify that I have answered every question and have not altered the wording of any of the questions on this form.

ICMJE DISCLOSURE FORM

**Date:**	1/15/2026
**Your Name:**	Joshua Grill
**Manuscript Title:**	Outreach, Recruitment and Engagement Cores of NIA-Designated Alzheimer's Disease Research Centers: Current Strategies and Future Directions
**Manuscript Number (if known):**	ADJ-D-25-03572

In the interest of transparency, we ask you to disclose all relationships/activities/interests listed below that are related to the content of your manuscript. "Related" means any relation with for-profit or not-for-profit third parties whose interests may be affected by the content of the manuscript. Disclosure represents a commitment to transparency and does not necessarily indicate a bias. If you are in doubt about whether to list a relationship/activity/interest, it is preferable that you do so.

The author's relationships/activities/interests should be defined broadly. For example, if your manuscript pertains to the epidemiology of hypertension, you should declare all relationships with manufacturers of antihypertensive medication, even if that medication is not mentioned in the manuscript.

In item #1 below, report all support for the work reported in this manuscript without time limit. For all other items, the time frame for disclosure is the past 36 months.

		**Name all entities with whom you have this relationship or indicate none (add rows as needed)**	**Specifications/Comments (e.g., if payments were made to you or to your institution)**
		Time frame: Since the initial planning of the work	
1	All support for the present manuscript (e.g., funding, provision of study materials, medical writing, article processing charges, etc.) No time limit for this item.	☐ None	
		NIA	
			Click the tab key to add additional rows.
		Time frame: past 36 months	
**2**	Grants or contracts from any entity (if not indicated in item #1 above).	☐ **None**	
		NIA, Alzheimer's Association, BrightFocus Fnd	
		Eli Lilly, Biogen, Genentech, Eisai	
**3**	Royalties or licenses	☒ **None**	

		Name all entities with whom you have this relationship or indicate none (add rows as needed)	Specifications/Comments (e.g., if payments were made to you or to your institution)
**4**	Consulting fees	☐ **None**	
		Valuate Health	
**5**	Payment or honoraria for lectures, presentations, speakers bureaus, manuscript writing or educational events	☒ **None**	
**6**	Payment for expert testimony	☒ **None**	
**7**	Support for attending meetings and/or travel	☐ **None**	
		Alzheimer's Association	
**8**	Patents planned, issued or pending	☒ **None**	
**9**	Participation on a Data Safety Monitoring Board or Advisory Board	☒ **None**	
**10**	Leadership or fiduciary role in other board, society, committee or advocacy group, paid or unpaid	☒ **None**	

		Name all entities with whom you have this relationship or indicate none (add rows as needed)	Specifications/Comments (e.g., if payments were made to you or to your institution)
**11**	Stock or stock options	☒ **None**	
**12**	Receipt of equipment, materials, drugs, medical writing, gifts or other services	☒ **None**	
**13**	Other financial or non-financial interests	☐ **None**	
		Travel covered by the Alzheimer's Association	

Please place an "X" next to the following statement to indicate your agreement:

☒ I certify that I have answered every question and have not altered the wording of any of the questions on this form.

ICMJE DISCLOSURE FORM

Date: 1/15/2026

Your Name: Crystal M Glover

Manuscript Title: Outreach, Recruitment and Engagement Cores of NIA-Designated Alzheimer's Disease Research Centers: Current Strategies and Future Directions

Manuscript Number (if known): ADJ-D-25-03572

In the interest of transparency, we ask you to disclose all relationships/activities/interests listed below that are related to the content of your manuscript. "Related" means any relation with for-profit or not-for-profit third parties whose interests may be affected by the content of the manuscript. Disclosure represents a commitment to transparency and does not necessarily indicate a bias. If you are in doubt about whether to list a relationship/activity/interest, it is preferable that you do so.

The author's relationships/activities/interests should be defined broadly. For example, if your manuscript pertains to the epidemiology of hypertension, you should declare all relationships with manufacturers of antihypertensive medication, even if that medication is not mentioned in the manuscript.

In item #1 below, report all support for the work reported in this manuscript without time limit. For all other items, the time frame for disclosure is the past 36 months.

		**Name all entities with whom you have this relationship or indicate none (add rows as needed)**	**Specifications/Comments (e.g., if payments were made to you or to your institution)**
		Time frame: Since the initial planning of the work	
1	All support for the present manuscript (e.g., funding, provision of study materials, medical writing, article processing charges, etc.) No time limit for this item.	☐ None	
		P30AG066519, MPI: LaFerla, Grill	Payments made to my institution for my percent effort as part of this grant.
		Department of Neurology, School of Medicine, UCI	Payments made to me as salary and other support as part of my faculty role.
		RF1NS143766, MPI: Flatt, Glover	Payments made to my institution for my percent effort as part of this grant.
		R01AG095017, MPI: Ezzati, Glover, Grill, Sajjadi	Payments made to my institution for my percent effort as part of this grant.
		Time frame: past 36 months	
**2**	Grants or contracts from any entity (if not indicated in item #1 above).	☐ **None**	
		NIH R01AG050782	I am a Co-Investigator on this grant.
		NIH **U24AG057437**	I am a Co-Investigator on this grant.
		NIH AG081811	I was a Co-Investigator on this grant.
		Memesto Small Business Innovation Research	I was a Co-Investigator on this grant.
		2R01AG022018-12	I was a Co-Investigator on this grant.
		R01AG062711	I was a Co-Investigator on this grant.
		R01AG060376	I was a Co-Investigator on this grant.
		**P30AG072975**	I was a Co-Investigator on this grant.

		Name all entities with whom you have this relationship or indicate none (add rows as needed)	Specifications/Comments (e.g., if payments were made to you or to your institution)
		U54AG063546	I am a Co-Investigator on this grant.
3	Royalties or licenses	☒ None	
4	Consulting fees	☐ None	
		Editor in Chief of Alzheimer's & Dementia: Behavior & Socioeconomics of Aging	Payments made to me.
5	Payment or honoraria for lectures, presentations, speakers bureaus, manuscript writing or educational events	☐ None	
		Dr. Solomon Carter Fuller Brain Health Summit, Wake Forest Alzheimer's Disease Research Center,	Honoraria made to me.
		Plenary Presenter, Plenary: Brain Health for All: Engaging Communities in Research, Education, and Treatment, American Association of Geriatric Psychiatry	Honoraria made to me.
		Social Activity: Engagement and Isolation." Dementia Risk Reduction and Social Determinant Webinar Series,	Honoraria made to me.
		Keynote Speaker, 2024 Arnold Aging Lecture, The Arnold School of Public Health, University of South Carolina	Honoraria made to me.
		Social Activity: Engagement and Isolation." Invited Speaker, Risk Reduction Subcommittee, The National Alzheimer's Project Act (NAPA)	Honoraria made to me.
		Distinguished Speaker, 2023 Alzheimer's Disease and Related Dementias Research Day, Alzheimer's Disease Research Center, University of Wisconsin, Madison, Madison, Wisconsin and Virtual, April 2023.	Honoraria made to me.
		Featured Speaker, Monthly Speaker Series, Virginia Alzheimer's Disease Center, University of Virginia	Honoraria made to me.
		Sixth Annual Medical, Scientific, and Memory Screening Advisory Board Roundtable, Alzheimer's Foundation of America (AFA),	Honoraria made to me.
		Indiana University Bloomington	Honoraria made to me.

		Name all entities with whom you have this relationship or indicate none (add rows as needed)	Specifications/Comments (e.g., if payments were made to you or to your institution)
**6**	Payment for expert testimony	☒ **None**	
**7**	Support for attending meetings and/or travel	☐ **None**	
		Alzheimer's Association	Support for Attending Meetings
		Wake Forest University	Support for Attending Meetings
		American Association of Geriatric Psychiatry (AAGP)	Support for Attending Meetings
		Alzheimer's Fast Track, BrightFocus Foundation	Support for Attending Meetings
		Indiana Alzheimer's Disease Research Center	Support for Attending Meetings
		University of South Carolina	Support for Attending Meetings
		Louisiana State University	Support for Attending Meetings
		University of California, Irvine	Support for Attending Meetings
		The University of Nevada, Las Vegas	Support for Attending Meetings
		Second Annual Black Men's Brain Health (BMBH) Conference,	Support for Attending Meetings
		Banner Alzheimer's Institute	Support for Attending Meetings
		University of Wisconsin, Madison	Support for Attending Meetings
**8**	Patents planned, issued or pending	☒ **None**	
**9**	Participation on a Data Safety Monitoring Board or Advisory Board	☐ **None**	
		Special Advisor, Independent Trial Steering Committee, Africa-FINGERS	Advisor, no payment
		Scientific Advisor, Hilarity for Charity	Advisor, no payment
		Scientific Program Committee (SPC), AAIC Advancements: Exploring Equity in Diagnosis Meeting, Alzheimer's Association	Ex-Officio Member, no payment
		The CaRE Course: **C**aregiving and **Ra**cial Considerations during a Pandemic Online Training **E**ducation, Retirement Research Foundation	External Advisory Board Member, no payment
		Alzheimer's Association, Orange County Chapter	Board Member, no payment
**10**	Leadership or fiduciary role in other board, society, committee or advocacy group, paid or unpaid	☐ **None**	
		Thresholds Psychiatric Rehabilitation Centers	Unpaid
		Ex-Officio Member, Scientific Program Committee (SPC), AAIC Advancements: Exploring Equity in Diagnosis Meeting, Alzheimer's Association	Unpaid

		Name all entities with whom you have this relationship or indicate none (add rows as needed)	Specifications/Comments (e.g., if payments were made to you or to your institution)
		Scientific Program Committee (SPC), Alzheimer's Association International Conference (AAIC)	Unpaid; Travel and Registration covered
**11**	Stock or stock options	☒ **None**	
**12**	Receipt of equipment, materials, drugs, medical writing, gifts or other services	☒ **None**	
**13**	Other financial or non-financial interests	☒ **None**	

Please place an "X" next to the following statement to indicate your agreement:

☒ I certify that I have answered every question and have not altered the wording of any of the questions on this form.

ICMJE DISCLOSURE FORM

**Date:**	1/19/2026
**Your Name:**	Darby Morhardt PhD, LCSW
**Manuscript Title:**	Outreach, Recruitment and Engagement Cores of NIA-Designated Alzheimer's Disease Research Centers: Current Strategies and Future Directions
**Manuscript Number (if known):**	ADJ-D-25-03572

In the interest of transparency, we ask you to disclose all relationships/activities/interests listed below that are related to the content of your manuscript. "Related" means any relation with for-profit or not-for-profit third parties whose interests may be affected by the content of the manuscript. Disclosure represents a commitment to transparency and does not necessarily indicate a bias. If you are in doubt about whether to list a relationship/activity/interest, it is preferable that you do so.

The author's relationships/activities/interests should be defined broadly. For example, if your manuscript pertains to the epidemiology of hypertension, you should declare all relationships with manufacturers of antihypertensive medication, even if that medication is not mentioned in the manuscript.

In item #1 below, report all support for the work reported in this manuscript without time limit. For all other items, the time frame for disclosure is the past 36 months.

		**Name all entities with whom you have this relationship or indicate none (add rows as needed)**	**Specifications/Comments (e.g., if payments were made to you or to your institution)**
**Time frame: Since the initial planning of the work**			
1	All support for the present manuscript (e.g., funding, provision of study materials, medical writing, article processing charges, etc.) No time limit for this item.	☐ None	
		NIH/NIA<br>P30 AG072977-01<br>Northwestern University Alzheimer's Disease Research Center	
			Click the tab key to add additional rows.
**Time frame: past 36 months**			
**2**	Grants or contracts from any entity (if not indicated in item #1 above).	☒ **None**	
**3**	Royalties or licenses	☒ **None**	

		Name all entities with whom you have this relationship or indicate none (add rows as needed)	Specifications/Comments (e.g., if payments were made to you or to your institution)
**4**	Consulting fees	☒ **None**	
**5**	Payment or honoraria for lectures, presentations, speakers bureaus, manuscript writing or educational events	☒ **None**	
**6**	Payment for expert testimony	☒ **None**	
**7**	Support for attending meetings and/or travel	☒ **None**	
**8**	Patents planned, issued or pending	☒ **None**	
**9**	Participation on a Data Safety Monitoring Board or Advisory Board	☒ **None**	
**10**	Leadership or fiduciary role in other board, society, committee or advocacy group, paid or unpaid	☐ **None**	
		Leadership and Service<br>2024- University of Illinois Chicago, Center for Health Equity in Cognitive Aging – Joining Population Science and Health Promotion RCMAR External Advisory Committee<br>2022- Appointment – Illinois Supreme Court Commission on Elder Law	

		Name all entities with whom you have this relationship or indicate none (add rows as needed)	Specifications/Comments (e.g., if payments were made to you or to your institution)
		Alzheimer's Disease Advisory Committee (ADAC) - Illinois Department of Public Health<br>2026 IL Department of Public Health – Alzheimer State Plan 2026-2029<br>2023 IL Department of Public Health – Alzheimer State Plan 2023-2026<br>2021-22ADAC Workforce Expansion & Training Taskforce<br>2021-22 ADAC Budget Planning Workgroup<br>2020 IL Department of Public Health – Alzheimer State Plan 2020-2023<br>2017 IL Department of Public Health – Alzheimer State Plan 2017-2020<br>2011, 2021 Alzheimer's and related disorders competency development<br>2000- ADAC committee member<br><br>Alzheimer's Association-IL Chapter<br>2023- Research Committee Member, 2024 Co-Chair Research Conference<br>2022- Committee Member, Release the Silence Annual African American Conference<br><br>2021 Faculty, Alzheimer's Association Interdisciplinary Summer Research Institute<br>2018- Faculty, Project ECHO for Dementia Diagnosis and Care: Primary Care Pilot, Alzheimer's Association<br><br>National Association of Social Workers (NASW)<br>2018-2025. Co-Chair-Shared Interest Group on Aging (NASW-IL)	
**11**	Stock or stock options	☒ **None**	
**12**	Receipt of equipment, materials, drugs, medical writing, gifts or other services	☒ **None**	

		Name all entities with whom you have this relationship or indicate none (add rows as needed)	Specifications/Comments (e.g., if payments were made to you or to your institution)
**13**	Other financial or non-financial interests	☒ **None**	

Please place an "X" next to the following statement to indicate your agreement:

☒ I certify that I have answered every question and have not altered the wording of any of the questions on this form.

ICMJE DISCLOSURE FORM

**Date:**	1/22/2026
**Your Name:**	Dorothy Farrar Edwards
**Manuscript Title:**	Outreach, Recruitment and Engagement Cores of NIA-Designated Alzheimer's Disease Research Centers: Current Strategies and Future Directions
**Manuscript Number (if known):**	ADJ-D-25-03572

In the interest of transparency, we ask you to disclose all relationships/activities/interests listed below that are related to the content of your manuscript. "Related" means any relation with for-profit or not-for-profit third parties whose interests may be affected by the content of the manuscript. Disclosure represents a commitment to transparency and does not necessarily indicate a bias. If you are in doubt about whether to list a relationship/activity/interest, it is preferable that you do so.

The author's relationships/activities/interests should be defined broadly. For example, if your manuscript pertains to the epidemiology of hypertension, you should declare all relationships with manufacturers of antihypertensive medication, even if that medication is not mentioned in the manuscript.

In item #1 below, report all support for the work reported in this manuscript without time limit. For all other items, the time frame for disclosure is the past 36 months.

		**Name all entities with whom you have this relationship or indicate none (add rows as needed)**	**Specifications/Comments (e.g., if payments were made to you or to your institution)**
		Time frame: Since the initial planning of the work	
1	All support for the present manuscript (e.g., funding, provision of study materials, medical writing, article processing charges, etc.) No time limit for this item.	☐ None	
		Wisconsin Alzheimer's Disease Research Center NIA 2P30AG033514-02	University of Wisconsin Madison
			Click the tab key to add additional rows.
		Time frame: past 36 months	
**2**	Grants or contracts from any entity (if not indicated in item #1 above).	☒ **None**	
**3**	Royalties or licenses	☒ **None**	

		Name all entities with whom you have this relationship or indicate none (add rows as needed)	Specifications/Comments (e.g., if payments were made to you or to your institution)
**4**	Consulting fees	☒ **None**	
**5**	Payment or honoraria for lectures, presentations, speakers bureaus, manuscript writing or educational events	☒ **None**	
**6**	Payment for expert testimony	☒ **None**	
**7**	Support for attending meetings and/or travel	☒ **None**	
**8**	Patents planned, issued or pending	☒ **None**	
**9**	Participation on a Data Safety Monitoring Board or Advisory Board	☒ **None**	
**10**	Leadership or fiduciary role in other board, society, committee or advocacy group, paid or unpaid	☒ **None**	

		Name all entities with whom you have this relationship or indicate none (add rows as needed)	Specifications/Comments (e.g., if payments were made to you or to your institution)
**11**	Stock or stock options	☒ **None**	
**12**	Receipt of equipment, materials, drugs, medical writing, gifts or other services	☒ **None**	
**13**	Other financial or non-financial interests	☒ **None**	

Please place an "X" next to the following statement to indicate your agreement:

☒ I certify that I have answered every question and have not altered the wording of any of the questions on this form.

ICMJE DISCLOSURE FORM

Date: 1/15/2026

Your Name: Carol K Chan

Manuscript Title: Outreach, Recruitment and Engagement Cores of NIA-Designated Alzheimer's Disease Research Centers: Current Strategies and Future Directions

Manuscript Number (if known): ADJ-D-25-03572

In the interest of transparency, we ask you to disclose all relationships/activities/interests listed below that are related to the content of your manuscript. "Related" means any relation with for-profit or not-for-profit third parties whose interests may be affected by the content of the manuscript. Disclosure represents a commitment to transparency and does not necessarily indicate a bias. If you are in doubt about whether to list a relationship/activity/interest, it is preferable that you do so.

The author's relationships/activities/interests should be defined broadly. For example, if your manuscript pertains to the epidemiology of hypertension, you should declare all relationships with manufacturers of antihypertensive medication, even if that medication is not mentioned in the manuscript.

In item #1 below, report all support for the work reported in this manuscript without time limit. For all other items, the time frame for disclosure is the past 36 months.

		**Name all entities with whom you have this relationship or indicate none (add rows as needed)**	**Specifications/Comments (e.g., if payments were made to you or to your institution)**
Time frame: Since the initial planning of the work			
1	All support for the present manuscript (e.g., funding, provision of study materials, medical writing, article processing charges, etc.) No time limit for this item.	☐ None	
		P30 AG072959	Institution
			Click the tab key to add additional rows.
Time frame: past 36 months			
**2**	Grants or contracts from any entity (if not indicated in item #1 above).	☒ **None**	
**3**	Royalties or licenses	☒ **None**	

		Name all entities with whom you have this relationship or indicate none (add rows as needed)	Specifications/Comments (e.g., if payments were made to you or to your institution)
**4**	Consulting fees	☒ **None**	
**5**	Payment or honoraria for lectures, presentations, speakers bureaus, manuscript writing or educational events	☒ **None**	
**6**	Payment for expert testimony	☒ **None**	
**7**	Support for attending meetings and/or travel	☒ **None**	
**8**	Patents planned, issued or pending	☒ **None**	
**9**	Participation on a Data Safety Monitoring Board or Advisory Board	☒ **None**	
**10**	Leadership or fiduciary role in other board, society, committee or advocacy group, paid or unpaid	☒ **None**	

		Name all entities with whom you have this relationship or indicate none (add rows as needed)	Specifications/Comments (e.g., if payments were made to you or to your institution)
**11**	Stock or stock options	☒ **None**	
**12**	Receipt of equipment, materials, drugs, medical writing, gifts or other services	☒ **None**	
**13**	Other financial or non-financial interests	☒ **None**	

Please place an "X" next to the following statement to indicate your agreement: CKC

☒ I certify that I have answered every question and have not altered the wording of any of the questions on this form.

ICMJE DISCLOSURE FORM

**Date:**	1/15/2026
**Your Name:**	Ozioma C. Okonkwo
**Manuscript Title:**	Outreach, Recruitment and Engagement Cores of NIA-Designated Alzheimer's Disease Research Centers: Current Strategies and Future Directions
**Manuscript Number (if known):**	ADJ-D-25-03572

In the interest of transparency, we ask you to disclose all relationships/activities/interests listed below that are related to the content of your manuscript. "Related" means any relation with for-profit or not-for-profit third parties whose interests may be affected by the content of the manuscript. Disclosure represents a commitment to transparency and does not necessarily indicate a bias. If you are in doubt about whether to list a relationship/activity/interest, it is preferable that you do so.

The author's relationships/activities/interests should be defined broadly. For example, if your manuscript pertains to the epidemiology of hypertension, you should declare all relationships with manufacturers of antihypertensive medication, even if that medication is not mentioned in the manuscript.

In item #1 below, report all support for the work reported in this manuscript without time limit. For all other items, the time frame for disclosure is the past 36 months.

		**Name all entities with whom you have this relationship or indicate none (add rows as needed)**	**Specifications/Comments (e.g., if payments were made to you or to your institution)**
**Time frame: Since the initial planning of the work**			
1	All support for the present manuscript (e.g., funding, provision of study materials, medical writing, article processing charges, etc.) No time limit for this item.	☐ None	
		NIH	Grants to my institution
			Click the tab key to add additional rows.
**Time frame: past 36 months**			
**2**	Grants or contracts from any entity (if not indicated in item #1 above).	☒ **None**	
**3**	Royalties or licenses	☒ **None**	

		Name all entities with whom you have this relationship or indicate none (add rows as needed)	Specifications/Comments (e.g., if payments were made to you or to your institution)
**4**	Consulting fees	☒ **None**	
**5**	Payment or honoraria for lectures, presentations, speakers bureaus, manuscript writing or educational events	☒ **None**	
**6**	Payment for expert testimony	☒ **None**	
**7**	Support for attending meetings and/or travel	☒ **None**	
**8**	Patents planned, issued or pending	☒ **None**	
**9**	Participation on a Data Safety Monitoring Board or Advisory Board	☒ **None**	
**10**	Leadership or fiduciary role in other board, society, committee or advocacy group, paid or unpaid	☐ **None**	
		International Neuropsychological Society	Treasurer

		Name all entities with whom you have this relationship or indicate none (add rows as needed)	Specifications/Comments (e.g., if payments were made to you or to your institution)
**11**	Stock or stock options	☒ **None**	
**12**	Receipt of equipment, materials, drugs, medical writing, gifts or other services	☒ **None**	
**13**	Other financial or non-financial interests	☒ **None**	

Please place an "X" next to the following statement to indicate your agreement:

☒ I certify that I have answered every question and have not altered the wording of any of the questions on this form.

ICMJE DISCLOSURE FORM

Date: 1/20/2026

Your Name: Monica Parker MD

Manuscript Title: Outreach, Recruitment and Engagement Cores of NIA-Designated Alzheimer's Disease Research Centers: Current Strategies and Future Directions

Manuscript Number (if known): ADJ-D-25-03572

In the interest of transparency, we ask you to disclose all relationships/activities/interests listed below that are related to the content of your manuscript. "Related" means any relation with for-profit or not-for-profit third parties whose interests may be affected by the content of the manuscript. Disclosure represents a commitment to transparency and does not necessarily indicate a bias. If you are in doubt about whether to list a relationship/activity/interest, it is preferable that you do so.

The author's relationships/activities/interests should be defined broadly. For example, if your manuscript pertains to the epidemiology of hypertension, you should declare all relationships with manufacturers of antihypertensive medication, even if that medication is not mentioned in the manuscript.

In item #1 below, report all support for the work reported in this manuscript without time limit. For all other items, the time frame for disclosure is the past 36 months.

		Name all entities with whom you have this relationship or indicate none (add rows as needed)	Specifications/Comments (e.g., if payments were made to you or to your institution)
**Time frame: Since the initial planning of the work**			
1	All support for the present manuscript (e.g., funding, provision of study materials, medical writing, article processing charges, etc.) **No time limit for this item.**	☑ **None**	
			Click the tab key to add additional rows.
**Time frame: past 36 months**			
2	Grants or contracts from any entity (if not indicated in item #1 above).	☑ **None**	
		Alzheimers Foundation of America	Payment To Emory
3	Royalties or licenses	☑ **None**	

[signature] 1/21/2026

		Name all entities with whom you have this relationship or indicate none (add rows as needed)	Specifications/Comments (e.g., if payments were made to you or to your institution)
4	Consulting fees	☑ None	
5	Payment or honoraria for lectures, presentations, speakers bureaus, manuscript writing or educational events	☐ None	
		Eisai	Pay Me
		Alz Association	To Me
		Lilly, Eli - Medical Education Advisory Group	To Me
6	Payment for expert testimony	☑ None	
7	Support for attending meetings and/or travel	☑ None	
8	Patents planned, issued or pending	☑ None	
9	Participation on a Data Safety Monitoring Board or Advisory Board	☑ None	
10	Leadership or fiduciary role in other board, society, committee or advocacy group, paid or unpaid	☐ None	
		Alzheimers Foundation of America -Chair Memory Screening & Scientific Advisory Board	Unpaid

[illegible]
1/21/2026

		Name all entities with whom you have this relationship or indicate none (add rows as needed)	Specifications/Comments (e.g., if payments were made to you or to your institution)
**11**	Stock or stock options	☑ **None**	
**12**	Receipt of equipment, materials, drugs, medical writing, gifts or other services	☑ **None**	
**13**	Other financial or non-financial interests	☐ **None**	
		AAFP- Commission on Continuin Professional Development	Unpaid

Please place an "X" next to the following statement to indicate your agreement:

☒ I certify that I have answered every question and have not altered the wording of any of the questions on this form.

[illegible signature] 1/21/2026

ICMJE DISCLOSURE FORM

**Date:**	1/15/2026
**Your Name:**	Sophia Wang
**Manuscript Title:**	Outreach, Recruitment and Engagement Cores of NIA-Designated Alzheimer's Disease Research Centers: Current Strategies and Future Directions
**Manuscript Number (if known):**	ADJ-D-25-03572

In the interest of transparency, we ask you to disclose all relationships/activities/interests listed below that are related to the content of your manuscript. "Related" means any relation with for-profit or not-for-profit third parties whose interests may be affected by the content of the manuscript. Disclosure represents a commitment to transparency and does not necessarily indicate a bias. If you are in doubt about whether to list a relationship/activity/interest, it is preferable that you do so.

The author's relationships/activities/interests should be defined broadly. For example, if your manuscript pertains to the epidemiology of hypertension, you should declare all relationships with manufacturers of antihypertensive medication, even if that medication is not mentioned in the manuscript.

In item #1 below, report all support for the work reported in this manuscript without time limit. For all other items, the time frame for disclosure is the past 36 months.

		**Name all entities with whom you have this relationship or indicate none (add rows as needed)**	**Specifications/Comments (e.g., if payments were made to you or to your institution)**
		**Time frame: Since the initial planning of the work**	
1	All support for the present manuscript (e.g., funding, provision of study materials, medical writing, article processing charges, etc.) No time limit for this item.	☐ None	
		K23AG062555	My institution
		P30AG072976	My institution
		R21AG074179	My institution
		R01AG084754	My institution
		**Time frame: past 36 months**	
**2**	Grants or contracts from any entity (if not indicated in item #1 above).	☒ **None**	
**3**	Royalties or licenses	☐ **None**	
		American Psychiatric Publishing Inc., book royalties	To me

		Name all entities with whom you have this relationship or indicate none (add rows as needed)	Specifications/Comments (e.g., if payments were made to you or to your institution)
**4**	Consulting fees	☒ **None**	
**5**	Payment or honoraria for lectures, presentations, speakers bureaus, manuscript writing or educational events	☒ **None**	
**6**	Payment for expert testimony	☒ **None**	
**7**	Support for attending meetings and/or travel	☒ **None**	
**8**	Patents planned, issued or pending	☒ **None**	
**9**	Participation on a Data Safety Monitoring Board or Advisory Board	☐ **None**	
		DSMB consultant fees for NIA funded grants	To me
**10**	Leadership or fiduciary role in other board, society, committee or advocacy group, paid or unpaid	☒ **None**	

		Name all entities with whom you have this relationship or indicate none (add rows as needed)	Specifications/Comments (e.g., if payments were made to you or to your institution)
**11**	Stock or stock options	☒ **None**	
**12**	Receipt of equipment, materials, drugs, medical writing, gifts or other services	☒ **None**	
**13**	Other financial or non-financial interests	☒ **None**	

Please place an "X" next to the following statement to indicate your agreement: SW

☒ I certify that I have answered every question and have not altered the wording of any of the questions on this form.
